# Gun-Shot Wounds of the Chest

**Published:** 1918-08

**Authors:** John Rose Bradford


**Gun Shot Wounds of the Chest as Seen in Hospitals on the Lines of Communication.** By Major General Sir John Rose Bradford.

Major General Bradford said in part :

To the physician the main problem in the treatment of these cases is that of forming an accurate diagnosis : first, as to the exact anatomical condition present; and second, as to the presence or absence of infection. For the adequate treatment of these cases it is essential that these patients should be placed in special wards under the care of a physician and a surgeon working together in close cooperation. Gunshot wounds of the chest are usually divided into two classes — the non-penetrating and the penetrating, but it must be remembered that it is by no means always a simple matter to form a correct opinion on any given case. Thus a penetrating or even a through and through wound of the chest may give rise to no appreciable lesion; and non-penetrating wounds may cause such effects as hemoptysis, hemothorax, massive collapse of the lung, pleurisy and pneumonia, most of which are more closely associated with penetrating wounds, and are therefore often regarded as affording conclusive evidence of the penetration of the missile. Further, an apparently trivial wound of some distant part of the body may be the point of entry of a missile that has really penetrated to the chest. Thus hemothorax and even pneumothorax may occur in consequence not only of wounds of the abdomen, but also of wounds of the face, neck, arm, and even, in rare instances, of the buttocks. Again even when the wound of entry is in the chest, the missile may pass through one side of the chest without causing any appreciable lesion on that side and yet cause a hemothorax, or a pneumothorax, on the opposite side.

In other instances, bilateral lesions such as bilateral hemothorax are produced : in such cases both effusions may be sterile, or both infected, or even one sterile and the other infected.

*Diagnosis.* As clearly mentioned there are two problems in diagnosis : (1) the exact nature of the lesion present, and (2) the presence or absence of infection.

The anatomical diagnosis must be made on careful study of the physical signs, and verified by exploratory puncture in cases of doubt.

*Physical Signs of Hemothorax.* — In a sterile or non-infected hemothorax, the chest on the affected side is immobile, often if not always retracted, the heart's apex beat is displaced, and the diaphragm on the affected side is displaced upwards and is immobile. This displacement of the dome of the diaphragm is a sign of considerable importance and is very characteristic of wounds of the chest where either hemothorax or massive collapse is present. Skodaic resonance is an extremely well marked phenomenon in hemothorax. On auscultation, weak breath sounds may be heard, but in many cases tubular or amphoric breathing is very well marked and bronchophony and whispering pectoriloquy are very obvious. In cases of moderate hemothorax the signs, therefore, are very liable to be confounded with those usually regarded as characteristic of consolidations; and a common error is thus made of thinking the patient is suffering from pneumonia, whereas, in reality, a bloody effusion is present in the chest. In cases where the hemothorax is very large in amount the signs present may approximate more closely to those characteristic of ordinary pleural effusion.

In exploratory puncture of the chest in cases of hemothorax, it is essential that the puncture should be made high up and not, as in ordinary pleural effusion, low down. This is necessary on account of the abnormally high position of the diaphragm in hemothorax. If this rule is not folloved, grave errors of diagnosis will be made.

In infected hemothorax the physical signs may at first be very silimar to those present in a sterile case, but they tend to increase and to alter rapidly and approximate to those of ordinary pleural effusion. Thus the retraction of the side is replaced by bulging with increased displacement of the heart and the development of increased pain and local tenderness on the affected chest. A more or less sudden and rapid increase in the effusion is in itself strong evidence of infection, but the most characteristic signs are the occurrence of a cracked pot percussion note over what was previously a dull area, and a sudden increase in the degree of displacement of the heart. These signs are due to the development of gas in the pleural cavity as a result of the presence of infection.

*Pneumothorax.* Pneumothorax is not very common and several varieties of the condition occur. In one form there is a gaping or sometimes a sucking wound of the chest wall; in the former case, the wound in the chest wall may be very large. Two points of interest may be mentioned with reference to these cases. In the first place, the lung in such instances does not usually collapse completely; it may retain approximately the cadaveric volume and this notwithstanding that the hole in the chest wall may be very large— i. e., admit the hand. Secondly, the great distress often present is relieved by the closure of the chest wall wound. In the other variety of pneumothorax, the so-called closed pneumothorax, the lung is usually completely collapsed. This variety of pneumothorax may be sterile or infected, and the latter is by far the most important from the point of view of treatment, as it is essential to recognize its presence without delay and to treat it by opening and cleansing the pleural cavity. A common error of diagnosis is that of regarding an infected pneumothorax as merely an increasing sterile one, i. e., to attribute the patient's distress to a mechanical condition when it is really mainly due to a toxic state. In some cases of infected pneumothorax the pneumothorax is localized and gas is not present in the whole extent of the pleural cavity. This localization is due to the presence of adhesions and in such cases there is usually great displacement of the heart and much distress.

*Massive Collapse of the Lung.* Collapse of the lung without any hemothorax or pneumothorax occurs in a certain but unknown proportion of gun shot wounds of chest, although it may also occur as a result of wounds of other parts of the body. It occurs not only in penetrating wounds of the chest but also in non-penetrating wounds and some of the most marked and extensive forms of massive collapse have been seen with non-penetrating wounds of the chest.

Collapse may be partial, lobar, or total. The partial form most usually involves the upper part of the lower lobe of one lung but not the apex of the lower lobe. The lobar variety affects usually the entire lower lobe, but cases have been seen where the upper lobe alone was involved. Collapse may occur on the same side as the lesion but there is a remarkable form in which it involves the lung on the side opposite to that wounded and where the wound is entirely unilateral. Some of the most marked examples of controlateral massive collapse have been in cases of quite trivial non-penetrating wounds of the chest wall.

The physical signs of massive collapse are very striking and characteristic. The chest wall is immobile and retracted, the heart is displaced towards the affected side, and the diaphragm is displaced upwards on the affected side. The signs present on auscultation may be divided into three periods :

In the first period there is either weakness or absence of breath sounds over the affected area. During the second stage tubular or even well marked amphoric breathing is heard, and during the third stage this abnoimal breathing is accompanied with rales and crepitations.

The physical signs resemble those of pneumonic consolidation; the retraction of the chest wall, the position of the heart and diaphragm give, however, the means of recognizing that collapse, and not pneumonic consolidation, is present. In some instances, as proved by post mortem findings, pneumonia and pleurisy occur as complications of massive collapse. Tbe signs of collapse may sometimes clear up in a few days; in other instances they may persist for as long as three weeks. Massive collapse may coexist with a small hemothorax and such cases often give rise to difficulty in diagnosis.

*Infected Hemothorax.* Infection of the pleural contents is present in some 25 per cent. of the cases of hemothorax and from the clinical study of the cases, five clinical types may be recognized ;

1. Severe and fulminating cases presenting symptoms that resemble those of secondary hemorrhage or of progressive pneumothorax.

2. Cases with symptoms of a milder type, but with severe, e.g., urgent dyspnea, tachycardia, pain; such cases may be mistaken for pneumonia owing to the fever, dyspnea, and bloodstained sputum.

3. Cases with severe symptoms suggestive of infection, but in which the bacteriological report states that the pleural fluid is sterile.

4. Cases with no urgent symptoms and with little or no pyrexia, but in which the pleural fluid contains organisms and has in some cases an offensive odor.

5. Cases of delayed infection. These cases run a course similar to that of non-infected cases, i. e., have no urgent symptoms for perhaps ten or fourteen days, and then suddenly urgent symptoms characteristic of infection occur.

*Complications.* True complications are rare except in infected hemothorax, but a number of conditions may be present in cases of gun shot wounds of the chest that for sake of brevity may be described as complications.

1. Associated injuries, more especially injuries of the spine and of the abdomen. Amongst the latter, wounds of the liver, stomach, and diaphragm are of special importance. Wounds and injuries of the diaphragm are of importance owing to the possible occurrence or later development of herniae.

2. Lesions of the pericardium, and especially hemopericardium, may occur in association with hemothorax and it may even occur with bilateral hemothorax and yet not be fatal. In rare instances a hemopericardium may be not only infected, but gas formation may take place leading to the production of a pneumopericardium. Two such cases are known to the writer that were successfully treated by drainage of the pericardium. True pericarditis is a common complication of infected hemothorax.

3. Aneurism of some large vessel in the neck may be present together with hemothorax or pneumothorax and one instance of traumatic aneurism of the thoracic aorta with hemothorax has fallen under the observation of the writer.

4. Bilateral and contralateral hemothorax are not very rare as a result of a single wound.

5. The most common complications of infected hemothorax are contralateral pleurisy and pneumonia, and pericarditis. Purulent bronchitis is also an important and serious complication.

6. Contralateral collapse may occur as a complication of both sterile and infected hemothorax and, as already mentioned, also in cases of non-penetrating wounds of the chest wall.

It is important to realize that pneumonia is rarely seen as a complication of gun shot wounds of the chest, and when it does occur it is usually in one of two conditions. First, as a complication of wounds of the chest wall without hemothorax where the infection spreads in from the infected wound tract; and, secondly, when it occurs as a contralateral complication of an infected hemothorax.

*Diagnosis of the Presence of Infection.* Three methods are available for this purpose.

1. *Clinical.* The character of the pyrexia and the type and severity of the symptoms are very useful means. The persistance of distressful dyspnea after the first day or two are suspicious signs and should always lead to the conclusion that infection is present. Furred tongue, delirium, and tachycardia are also of great importance, local tenderness over the sites of the affection is also suspicious. Jaundice is almost always a sign of infection, especially if marked. In the most severe cases jaundice is intense and may be accompanied by epistaxis and hematemesis. The signs to be relied upon are a rapid increase in the amount of the effusion and especially the development of the signs of the presence of gas in the pleura. The occurrence of complications such as pleurisy and pericarditis is also significant.

The most common errors are to impute the symptoms to secondary hemorrhage, to increasing pneumothorax, or to the supposed presence of pneumonia on the side of the hemothorax when really all the signs are due to toxemia from infection.

2. The examination of the fluid removed by an exploratory puncture is of great importance. Thus this fluid may show abundant polymorphs and also show evidence of marked hemolysis — both these facts are strong evidence of infection even if organisms are not seen. In some cases the foul odor will reveal infection even if organisms are not found.

3. In most cases bacteriological examination reveals the presence of organisms both in smear and culture, but in some cases none are found, although the clinical signs and the cell content of the pleural fluid both suggest the presence of infection. In such cases, where there is a conflict between the clinical and the bacteriological findings, repeated puncture at different levels should be practised, since in some instances the infection is limited by clotting and a focus of infection may be present in clot etc., whereas the main mass of hemothorax fluid is as yet free from infection. The decision in such cases is always difficult, but, if the patient's symptoms are severe, and there is no other cause than the hemothorax to account for them, it is best to regard the case as an infected one, and treat it accordingly, notwithstanding the absence of bacteriological proof.

*Treatment of Hemothorax.* If the hemothorax is sterile and small in amount no special treatment is required. Aspiration should be employed in all cases of moderate or large size. The fluid should be removed slowly. The oxygen replacement method is of great value, inasmuch as by this means all the bloody effusion can be removed at one sitting with the minimum discomfort to the patient, provided local anesthesia is employed. In the exceptional cases were clotting *en masse* has occurred the chest should be opened and the clot removed, the chest being then closed and subsequently aspiration practised if necessary.

In cases of infected hemothorax there should be no delay in the treatment of the case by surgical methods, such as drainage of the pleura or preferably by cleansing the pleura after the excision of a long length of rib with immediate closure of the chest and subsequent repeated aspiration.

The only class of case that admits of delay is that in which the patient presents no symptoms and yet the pleural fluid contains organisms. Some of these cases undoubtedly get well if only treated with aspiration, but in many of these the patient's condition is not really satisfactory, the fluid reaccumulates and it is questionable whether it is not better to treat such cases by opening and cleansing the pleura with immediate closure.

